# Stress and coping strategies among nursing students in clinical practice: a qualitative study*

**DOI:** 10.1590/1980-220X-REEUSP-2026-0056en

**Published:** 2026-10-12

**Authors:** Sara Soares dos Santos, Ítalo Rodolfo Silva, Agostinho A. C. Araújo, Simone de Godoy, Emerson Willian Santos de Almeida, Lucas Gardim, Kristina Mikkonen, Isabel Amélia Costa Mendes

**Affiliations:** 1 Universidade de São Paulo, Escola de Enfermagem de Ribeirão Preto, Ribeirão Preto, SP, Brazil.; 2 Universidade Federal do Rio de Janeiro, Escola de Enfermagem Anna Nery, Rio de Janeiro, RJ, Brazil.; 3 University of Oulu, Faculty of Medicine, Research Unit of Health Sciences and Technology, Oulu, Finland.

**Keywords:** Stress, Psychological, Education, Nursing, Students, Nursing

## Abstract

**Objective:**

To explore nursing students’ experiences with stress in clinical practice and the coping strategies they employ in that context.

**Method:**

A qualitative study based on *Grounded Theory* (GT) from the Corbinian perspective, conducted with 15 undergraduate nursing students at a public university in the Southeast region of Brazil.

**Results:**

For nursing students, the start of clinical practice was marked by stress, insecurity, and difficulties adapting, influenced by the organization of activities, the relationships established in clinical settings, and the available support. Coping strategies included: 1) individual actions, such as emotional regulation and seeking support; and 2) collective actions, such as peer support and institutional spaces for dialogue.

**Conclusion:**

Stress in clinical practice emerges as a challenging formative experience influenced by individual, relational, and institutional factors. Recognizing these dimensions broadens the possibilities for pedagogical and institutional intervention, contributing to the quality of education in the context of clinical practice and to students’ adaptation throughout their nursing education.

## INTRODUCTION

Students are a group that is particularly vulnerable to stressful experiences, and academic stress is recognized as a significant public health challenge due to its impact on mental health^(1)^. In the field of health education, nursing students experience higher levels of stress compared to students in other fields, primarily due to prolonged contact with patients during clinical practice^(2)^. Although clinical practice is essential for linking theoretical knowledge to practical application^(3)^, evidence suggests that this environment is often perceived as challenging and stressful^(3,4)^.

Among the main stressors identified in this context are uncertainty regarding theoretical and practical training, a strong sense of responsibility for patient care, the perception of inadequate skills, time pressure, and difficulties in interpersonal relationships^(5)^. These factors can compromise students’ quality of life, with negative repercussions on their personal lives and academic performance^(4)^.

Despite ongoing changes in health care systems and the growing complexity of clinical settings, there remains a limited understanding of how nursing students’ exposure to clinical practice is organized and structured. Clinical education still favors short-term, task-oriented approaches at the expense of preparing students for future challenges and changes in professional practice^(6)^. Although previous studies have sought to explore these issues^(7,8,9)^, many do not take an in-depth look at students’ subjective experiences. Given this, this study seeks to answer the following questions: What are nursing students’ experiences and perceptions regarding the clinical practice environment, and what coping strategies do they adopt to deal with the challenges in this context?

In this context, Grounded Theory (GT) is an appropriate approach for investigating such issues, as it values participants’ experiences and enables theoretical construction based on empirical data, thereby contributing to the development of strategies aimed at promoting well-being and improving academic performance^(10)^. Therefore, the purpose of this study is to explore nursing students’ experiences with stress in clinical practice and the coping strategies they employ in that context.

## METHOD

### Study Type/Design

This was a qualitative study based on the GT methodological framework, from a Corbinian perspective, whose analytical process involves a comparative dynamic across different stages of the research process, positioning categories within a paradigmatic model that objectively facilitates understanding of the phenomenon under investigation^(11,12)^. This study was conducted in accordance with COREQ guidelines^(13)^, as part of an integrated research project on stress among nursing students during clinical practice.

### SETTING

The study was conducted at a public university located in the Southeast region of Brazil and involved two undergraduate nursing programs: the Bachelor of Nursing (eight semesters) and the Bachelor’s Degree in Nursing with Teaching Licensure (ten semesters). Both programs aim to train nurses to work in various healthcare settings. However, in addition to training nurses, the second program adds two semesters and qualifies students to teach in professional nursing education. In accordance with the principles of GT, the inclusion of the two programs was based on the hypothesis that context can influence students’ experiences and perceptions of clinical practices, as well as their coping strategies with stress.

### Population and Selection Criteria

Students from the second year of the program onward participated; this is the period when clinical rotations in public health services begin. Students on medical leave were excluded. There were no refusals, withdrawals, or declared conflicts of interest related to the study setting or the researchers involved.

### Data Collection

Semi-structured interviews were conducted between December 2023 and January 2024. It is worth noting that the data collection timeline was facilitated by the virtual and dynamic approach to participant recruitment, described below. Furthermore, the researchers who conducted the interviews (SSS and AACA) received training from a senior researcher with experience in qualitative research (IRS). Therefore, while one researcher coordinated the interview, the other took notes (field notes), observing the interviewees’ reactions and recording their own impressions as a researcher. These records were later incorporated into the analysis, serving to support the researchers during the coding and traceability stages of the concept-building process, as well as to identify theoretical saturation of the data.

The first participant was selected for convenience to conduct a pilot interview, intended to test and refine the data collection protocol. After this stage, the instrument was adjusted, and the pilot-phase participant was not included in the study’s final sample. Based on this participant’s recommendations, other potential participants who met the established eligibility criteria were identified. Invitations were sent via WhatsApp, and subsequently, each participant referred new potential interviewees. Consequently, the sample was selected on a convenience basis, guided by the previously defined inclusion criteria. To characterize the participants, a sociodemographic questionnaire was administered (including gender, date of birth, undergraduate major, and years of education); subsequently, to define the analytical scope, semi-structured interviews were conducted, with guiding questions developed based on a scoping review^(14)^. Among the questions identified, the following stood out as the ones that guided the interviews: 1) *Please tell me about your experience in a clinical setting*; 2) *What do you understand by stress?* In addition, circular questions were asked to explore the topic in greater depth. The interviews were conducted via videoconference using the freely accessible Zoom Workplace platform, were audio-recorded, and lasted an average of 35 minutes. The information provided was verified against the curricular and pedagogical frameworks of the two courses to ensure data reliability.

It is worth noting the researchers’ epistemological stance of not subjecting the interviews to content validation by the interviewees, as it is in the nature of GT that the data originate from the investigative process during data collection. Thus, additional information arising from subsequent reflections could introduce bias into the research.

The interviews were concluded upon reaching theoretical saturation of the data, a point observed and discussed by the researchers based on the understanding that the concepts had been thoroughly developed. In research conducted using GT, this process is facilitated by the simultaneity of data collection and analysis and supported by memos prepared during the analysis. Theoretical saturation was analyzed, discussed, and determined by two researchers (SSS and IRS).

### Data Analysis and Processing

The interviews were transcribed using MacWhisper. Additionally, to ensure reliability, these interviews were reviewed manually. Following the principles of GT, data processing and analysis occurred simultaneously with each interview, enabling successive cycles of continuous coding and constant comparison between data collection and analysis. This approach allows researchers to identify the development of concepts through the constant comparison of data.

In this regard, it is worth highlighting the analytical steps used to develop categories/concepts and their respective subcategories/principles, namely: open coding; axial coding; and selective integration^(11,12)^. Open coding began with microanalysis (line-by-line analysis of the interviews), a stage in which the data were segmented into distinct parts by identifying their properties and dimensions. By “properties and dimensions,” we mean the specific characteristics of the information that exhibit nuances of distinct qualities, intensities, and/or depths, even though they pertain to responses to questions that are common to a given sample group. This process resulted in the identification of preliminary codes through the grouping of data based on similarities in properties and dimensions.

Next, the preliminary codes were grouped, leading to the development of conceptual codes. Conceptual codes are broader, less descriptive elements that offer greater analytical depth, though still in a fragmented manner. From this point on, the axial coding stage began through a comparative analysis of the conceptual codes, during which the researchers developed subcategories that serve as more dense explanatory units regarding the reality under study and form principles that will come to constitute concepts/categories. However, the grounded nature of the method requires that the subcategories/principles revolve around a category—as indicated—whose explanatory density encompasses all the elements described in the set of conceptual codes that constituted the subcategories^(11,12)^.

In integrative coding, the categories were positioned within a paradigmatic model that allowed for greater coherence among them, thereby providing explanatory depth to the developed theory. From a Corbinian perspective, the elements of the paradigmatic model are: causes (factors that lead to the occurrence of the phenomenon under investigation); actions-interactions (intervention strategies—coping with stress, in the context of this study); and possible expected consequences (based on the established actions-interactions)^(11,12)^.

### Ethical Considerations

All participants signed an informed consent form prior to data collection. The study was approved by a Research Ethics Committee (CEP) under approval number CAAE: 69618723.9.0000.5393. The interviews, as well as the transcripts, were stored in a restricted folder on Google Drive, with access limited solely to the researchers involved. To ensure anonymity, the interviewees, as well as their data, were identified using the letter “S,” standing for “student,” followed by the respective numerical order of the interview (S01, S02, S03…), along with the course categorization “B” for Bachelor’s degree and “BT” for Bachelor’s and Teaching degree, in addition to the indication of the year of study.

## RESULTS

Based on eligibility criteria and theoretical saturation, 15 students were divided into two sample groups: seven from the Bachelor of Nursing program and eight from the Bachelor’s Degree in Nursing with Teaching Licensure program. The students were between 21 and 30 years old (mean age of 23) and were in their third through fifth years of study. In this regard, the theoretical construct titled “Transformations in Clinical Practice” explains contextual factors related to the stress experienced during clinical practice and informs actions and interventions tailored to the needs of nursing students (Chart 1).

**Chart 1:** Categories and subcategories – Ribeirão Preto, SP, Brazil, 2024.

Category	Subcategory
3.1 Contextual factors contributing to stress in the clinical practice of nursing students	3.1.1 Starting clinical practice: insecurity and uncertainty
	3.1.2 The influence of professors on the clinical practice experience
	3.1.3 The influence of senior students on expectations regarding clinical practice
3.2 Strategies for coping with stress among nursing students	3.2.1 Individual strategies for coping with stress
	3.2.2 Collective strategies for coping with stress

For this article, given the depth of the results, we present the categories that constitute the causal component based on contextual factors in nursing students’ clinical practice, and the actions-interactions component, through the identification of coping strategies for the stress experienced by nursing students in the context of clinical practice.

For students, the transition from a theoretical setting to clinical practice triggers an initial stress response. However, according to the participants, when accompanied by coping strategies, this transition facilitates adaptation; when it is not, it can result in physical and mental repercussions. Figure 1 illustrates this process of stress progression and the coping strategies identified.

**Figure 1:**
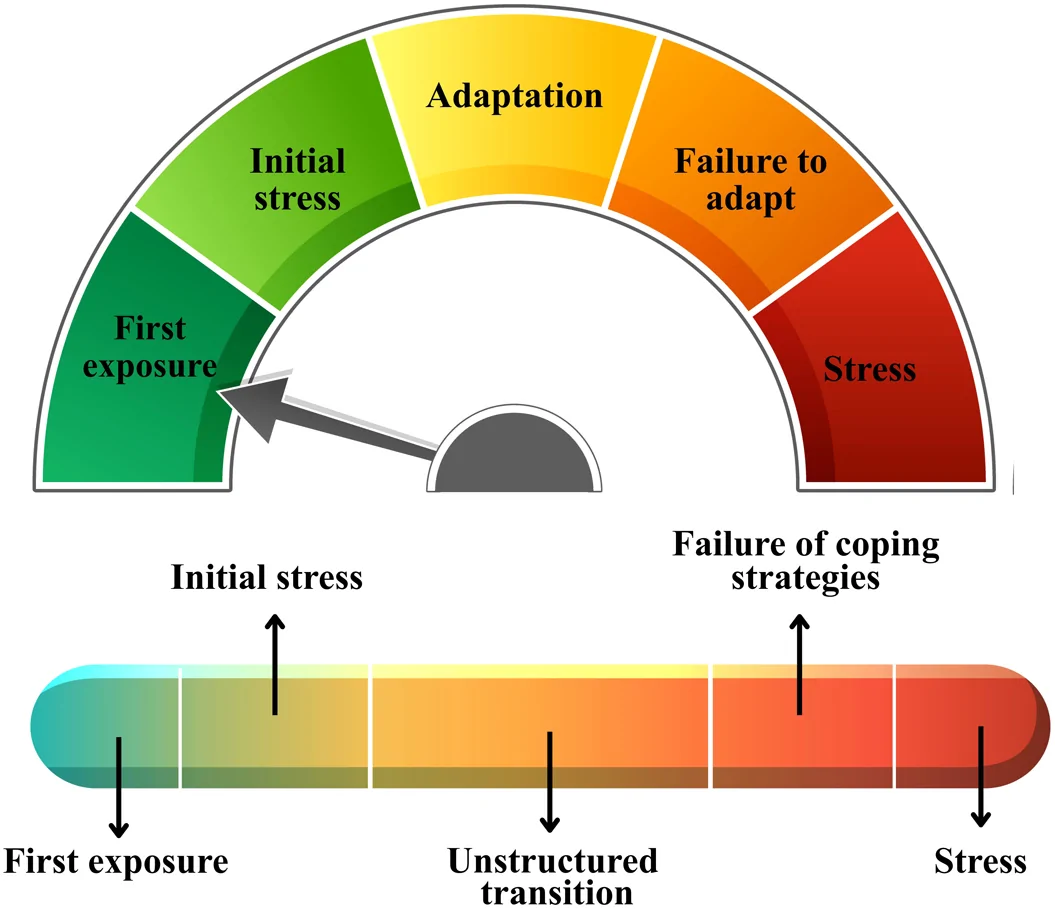
Nursing students’ experiences and coping strategies in clinical practice.

### Contextual Factors Contributing to Stress in the Clinical Practice of Nursing Students

This category comprises three subcategories that illustrate how context influences the onset, development, and experience of clinical practice, without a fixed sequence, depending on stress conditions and the coping strategies adopted. It should be noted that the excerpts from the testimonies presented are merely illustrative of the subcategories, as they result from an in-depth comparative analysis of various interviews.

#### Starting Clinical Practice: Insecurity and Uncertainty

Early experiences in clinical practice were marked by fear, insecurity, and uncertainty, stemming from the fear of harming patients, inexperience, and difficulty in decision-making.

*“I was really worried about my performance as a professional—I was genuinely afraid that I wouldn’t be able to give patients the attention and support they needed. Even though I was a student and knew I was prone to making mistakes [...] I always want what’s best for the patient and for the people on my team, and I end up putting a lot of pressure on myself.”* (S02, BT, 3rd year)

The transition from the laboratory to real-world care caused anxiety, which was exacerbated by a heavy workload, the need to juggle multiple demands simultaneously, and the fear of making mistakes.

*“I think the sheer volume of activities plays a role, too, because there’s a lot to worry about—there are so many other tasks within the subject itself, right? [...] Each subject has its own unique aspects, so there are a lot of details you need to pay attention to, and they’re also tested in practice.”* (S01, B, 3rd year)

Advance preparation helped reduce the initial stress.

*“That guidance in those other subjects—I think it made us feel more confident and at ease, and it eased some of the stress of showing up on the first day and not knowing anything, of not having that support, of not having that network, you know?”* (S04, B, 3rd year)

The adjustment took place gradually, in tandem with the development of trust and a sense of support.

*“But as the days went by, I started to feel a little more secure, because I think the main thing is that I wasn’t alone, you know?”* (S09, BT, 5th year)

#### The Influence of Professors on the Clinical Practice Experience

Participants highlighted the influence of professors as a key factor in their clinical practice experience. Approaches that fostered autonomy, participation, and constructive feedback were associated with positive experiences.

*“There were some clinical rotations that I really enjoyed, because of the way the professor let us work during the rotation, and there were others that I really didn’t like. I think that had a direct impact on the experience.”* (S01, B, 3rd year)

*“I understand that supervision is necessary, but there were professors who gave us a lot more freedom and leeway to do things, perform procedures, and so on. And there were others who didn’t trust us and would just do it themselves, you know? They didn’t give students the chance to do it; they preferred to do it themselves and have the students watch.”* (S01, B, 3rd year)

According to the students, the professors’ support reduced their fear and boosted their confidence.

*“And I feel like she made me feel much more at ease, prepared, and confident to perform that part of the procedure. And she helped ease that fear I had—‘I’m going to kill the patient; I’m still a student, and I won’t be able to do anything.’ [...] I felt very confident moving forward with the more hands-on part.”* (S15, B, 3rd year)

Warm and welcoming environments fostered emotional security, while authoritarian attitudes heightened anxiety.

*“When teachers are kind and understand that we are students who are still learning and developing our skills, we feel much freer and learn much more.*” (S08, B, 3rd year)

#### The Influence of Senior Students on Expectations Regarding Clinical Practice

The data indicated that accounts from senior students influenced expectations before the practicum began, especially when linked to negative experiences.

*“I think there’s a lot of prejudice here at school regarding this subject. We’ve heard from senior students [...] that it’s a difficult subject, that we’re not well-received, and that we have to put up with a lot.”* (S04, B, 3rd year)

*“I was really scared to start the clinical rotation, especially since it’s a course that people talk about a lot*.” (S11, B, 3rd year)

*“At first, I was a little apprehensive, because I’d heard a lot from senior students saying that the professors are, well, a little strict when it comes to internships, and they really are very strict. But I had an excellent experience—I didn’t have any bad experiences with the professors.”* (S02, BT, 3rd year)

Despite this, some participants reported positive experiences that exceeded their initial expectations.

“[...] *It was much calmer than people said*.” (S15, B, 3rd year)

### Strategies for Coping with Stress among Nursing Students

The students identified individual and collective strategies for coping with the stress arising from clinical practice and suggested institutional support measures, as highlighted in the subcategories below.

#### Individual Strategies for Coping with Stress

Physical activity, relaxation techniques, and support networks were described as essential for managing stress.

*“I think it’s really important to talk to our support network, you know? Friends or teachers. And, from there, identify key aspects that can help us deal with the situation and cope with stress as best as we can.”* (S06, BT, 5th year)

The support of professors and healthcare professionals fostered a sense of safety and belonging.

*“Being able to talk, one-on-one, without that sense of hierarchy, you know? Like teacher and student. Being able to express how you felt at a certain moment and having someone who helps you as much as they can.”* (S07, B, 3rd year)

Psychological counseling has also been recognized as an important resource for stress management.

#### Collective Strategies for Coping with Stress

Discussion groups and communal spaces were considered important for sharing experiences, strengthening bonds, and reducing stress.

*“Creating a healthy environment—a truly sustainable community environment—is very important so that these students feel at ease and, as a result, don’t feel stressed in situations that don’t require stress.”* (S05, BT, 3rd year)

The participants emphasized the importance of involving administrators, professors, and staff in spaces for dialogue.

*“I think it would be up to the management team there to organize a discussion with the staff members who could truly understand what’s really causing so much stress among the staff.”* (S02, BT, 3rd year)

*“I think that bringing the team together to discuss the strengths and weaknesses of the service and of each person’s practices also ends up enhancing everyone’s clinical practice.”* (S09, BT, 5th year)

Institutional strategies were suggested, such as adjusting the course load, gradual integration into clinical practice, crisis management protocols, individual and group mentoring, as well as simulations and *debriefings*.

*“I think it would be nice if we had something in our undergraduate program—I don’t know, maybe a group or even a class—that would help us have some conversations about how to deal with stress, you know?”* (S13, BT, 5th year)

Clinical experience was found to be influenced by the environment, interpersonal relationships, and professors’ attitudes, in conjunction with expectations and prior experiences.

## DISCUSSION

Clinical practice is an essential component of nursing education, enabling students to apply theoretical knowledge, develop skills, and understand the dynamics of professional practice^(6,15)^. This period is marked by anxiety, stress, and the need to adapt to an unfamiliar and demanding environment. Such experiences, which are common when faced with the unknown, tend to diminish as the adaptation process unfolds. However, if not properly managed, stress can reach harmful levels^(16)^.

Prior familiarity with the clinical setting is essential, since the environment influences the development of professional identity and is associated with moderate levels of stress during clinical rotations^(15,17)^. Students’ expectations, shaped by accounts from senior peers and the actions of educators, directly influence their perception of stress and the adaptation process, underscoring the importance of educational interventions that foster realistic expectations in order to break down stigmas that may exacerbate anxiety and, consequently, stress during clinical practice^(18)^. The role of the educator is central to the clinical practice experience, since teaching strategies, forms of interaction, and levels of support directly impact students’ satisfaction, learning, and engagement^(8,9,19)^. Collaborative and welcoming approaches foster autonomy, emotional security, and a sense of belonging, while overly controlling attitudes can limit participation and increase insecurity^(9)^.

Studies show that positive experiences and greater satisfaction are associated with effective stress-coping strategies, while the year of study also influences this process: students in higher years tend to approach problem-solving more efficiently^(20,21)^. In this context, the results of this study indicate that students used both individual and collective strategies to cope with stressors. Among the individual strategies, the following stand out: cognitive restructuring, social support, wellness practices (exercise, breathing exercises, music, reading), psychological counseling, and task focus.

On the other hand, passive coping styles, such as avoiding, postponing, or dealing with problems on one’s own, are associated with higher stress levels and only provide temporary relief, which can compromise one’s ability to handle critical situations^(20)^. In contrast, optimistic attitudes lead to lower stress levels and the use of problem-solving strategies, which are generally supported by peers, family members, and educators^(15)^. In addition, well-being practices, such as physical exercise, although classified by some studies as transfer strategies with limited effectiveness, are associated with emotional intelligence, self-efficacy, and emotional regulation^(22)^. Considering that self-efficacy contributes to better stress management and clinical performance^(20)^, such practices may promote emotional well-being, although further studies are needed to confirm their effectiveness. Social support emerged as a protective factor, especially when mediated by the active participation of educators^(23,24)^. Collective strategies, such as discussion groups, debriefings, and institutional spaces for dialogue, were identified as desirable for sharing experiences, strengthening bonds, and reducing stress.

Stress management begins with preparing students for clinical practice through measures that reduce anxiety and build skills. Courses that address the causes and effects of stress and strengthen self-confidence, self-efficacy, and resilience are essential for tackling clinical challenges^(20,25)^. In this regard, the results underscore the need for structured initiatives focused on clinical preparation, stress management, and the promotion of well-being, including educational programs, tutorials, realistic simulations, and emotional regulation activities^(15,26,27,28,29)^. Programs based on mindfulness and social support networks have also been shown to provide significant benefits^(30)^. At the organizational level, collaborative efforts among educational institutions, health care services, educators, and clinical instructors are essential for creating welcoming clinical practice environments that are committed to nursing education, reducing stressors, and promoting learning^(8)^. The use of debriefings at the end of practical activities stands out as an important strategy for assessment, feedback, and improvement of the training process.

Taken together, the findings of this study indicate that stress is experienced individually by students but is produced, intensified, or mitigated by collective, relational, and institutional factors, thus constituting a co-produced phenomenon within the educational context. Initial stress does not emerge solely “from within” the student, but is constructed based on concrete conditions of clinical practice—such as the abrupt start of activities, the workload, and the way the internship is organized, intertwined with the interpersonal relationships established in learning settings, particularly the educator’s role and the meanings previously attributed to the practice through accounts from senior students.

Furthermore, the collective dimension of training shapes how stress is perceived and managed, taking into account the presence or absence of institutional spaces for listening, peer support, and opportunities to share experiences. This understanding broadens the interpretation of stress in clinical practice as a dynamic process influenced by training conditions and contributes to reflection on how different pedagogical and institutional arrangements can affect students’ adaptation throughout their nursing education.

## CONCLUSION

This study revealed that, for nursing students, clinical practice is a formative period marked by insecurity, discomfort, and stress, especially at the beginning of their clinical activities, when they are less familiar with the environment, the demands of patient care, and the relationships established in clinical settings. By exploring students’ experiences and perceptions, the results show that this stress does not manifest uniformly but is shaped by the pedagogical, relational, and institutional conditions in which clinical practice occurs.

The identification of individual and collective coping strategies broadens our understanding of how students deal with the challenges of clinical practice and highlights that stress management can foster safer adaptation processes throughout their training. In this sense, the study contributes by demonstrating that perceived stress is not a fixed or inevitable obstacle, but rather an experience that can be recognized and managed through educational interventions, social support, and institutional organization that is more responsive to students’ needs.

By integrating these dimensions, the findings offer insights into improving pedagogical and institutional practices that enhance the clinical experience, promote student retention and development, and support their educational journey across the various stages of nursing education. In addition, the findings can guide future research, particularly longitudinal studies that assess the impact of educational interventions on stress throughout nursing education.

## Data Availability

The entire dataset supporting the results of this study was published in the article itself.
