# Supplementary material for: Stress and coping strategies among nursing students in clinical practice: a qualitative study
Source: Rev Esc Enferm USP. 2026 Oct 12;60:e20260056. doi: 10.1590/1980-220X-REEUSP-2026-0056en (PMC13680046; doi:10.1590/1980-220X-REEUSP-2026-0056en)
Supplement: Translation

# Translation: Estresse e estratégias de enfrentamento de estudantes de enfermagem na prática clínica: estudo qualitativo*

**DOI:** 10.1590/1980-220X-REEUSP-2026-0056pt

**Authors:** Sara Soares dos Santos, Ítalo Rodolfo Silva, Agostinho A. C. Araújo, Simone de Godoy, Emerson Willian Santos de Almeida, Lucas Gardim, Kristina Mikkonen, Isabel Amélia Costa Mendes

**Affiliations:** 1 Universidade de São Paulo, Escola de Enfermagem de Ribeirão Preto, Ribeirão Preto, SP, Brasil.; 2 Universidade Federal do Rio de Janeiro, Escola de Enfermagem Anna Nery, Rio de Janeiro, RJ, Brasil.; 3 University of Oulu, Faculty of Medicine, Research Unit of Health Sciences and Technology, Oulu, Finland.

**Keywords:** Estresse Psicológico, Educação em Enfermagem, Estudantes de Enfermagem

## Abstract

**Objetivo:**

Explorar as experiências de estudantes de enfermagem em relação ao estresse na prática clínica e as estratégias de enfrentamento adotadas nesse contexto.

**Método:**

Estudo qualitativo, cujo referencial metodológico foi a *Grounded Theory* (GT), na perspectiva corbiniana, realizado com 15 estudantes de graduação em enfermagem de uma universidade pública da região Sudeste do Brasil.

**Resultados:**

Para os estudantes de enfermagem, o início da prática clínica foi marcado por estresse, insegurança e dificuldades de adaptação, influenciados pela organização das atividades, pelas relações estabelecidas nos cenários de prática e pelo suporte disponível. As estratégias de enfrentamento envolveram: 1) ações individuais, como regulação emocional e busca por apoio; e 2) ações coletivas, como suporte entre pares e espaços institucionais de diálogo.

**Conclusão:**

O estresse na prática clínica emerge como uma vivência formativa desafiadora influenciada por fatores individuais, relacionais e institucionais. Reconhecer essas dimensões amplia as possibilidades de intervenção pedagógica e institucional, contribuindo para a qualificação da educação no contexto da prática clínica e para a adaptação dos estudantes ao longo da formação em enfermagem.

## INTRODUÇÃO

Estudantes constituem um grupo particularmente vulnerável a experiências estressantes, sendo o estresse acadêmico reconhecido como um relevante desafio de saúde pública em razão de seus impactos na saúde mental^(1)^. No âmbito da formação em saúde, estudantes de enfermagem apresentam níveis mais elevados de estresse quando comparados aos de outras áreas, sobretudo em função do contato prolongado com pacientes durante a prática clínica^(2)^. Embora a prática clínica seja fundamental para articular o conhecimento teórico à aplicação prática^(3)^, evidências indicam que esse ambiente é frequentemente percebido como desafiador e gerador de estresse^(3,4)^.

Entre os principais estressores identificados nesse contexto, destacam-se a insegurança quanto à formação teórica e prática, o elevado senso de responsabilidade diante do cuidado ao paciente, a percepção de insuficiência de habilidades, a pressão do tempo e as dificuldades nas relações interpessoais^(5)^. Tais fatores podem comprometer a qualidade de vida dos estudantes, com repercussões negativas sobre a vida pessoal e o desempenho acadêmico^(4)^.

Apesar das transformações contínuas nos sistemas de saúde e da crescente complexidade dos ambientes clínicos, persiste uma compreensão limitada acerca da organização e estruturação da exposição dos estudantes de enfermagem à prática clínica. A educação clínica ainda privilegia abordagens de curto prazo, orientadas para tarefas, em detrimento da preparação para desafios futuros e mudanças na prática profissional^(6)^. Embora estudos prévios tenham buscado explorar essas questões^(7,8,9)^, muitos não contemplam de forma aprofundada as experiências subjetivas dos estudantes. Diante disso, este estudo busca responder às seguintes questões: quais são as experiências e percepções de estudantes de enfermagem em relação ao ambiente da prática clínica e quais estratégias de enfrentamento são adotadas para lidar com os desafios nesse contexto?

Nessa conjuntura, a *Grounded Theory* (GT) constitui uma abordagem adequada para investigar tais questões, ao valorizar as experiências dos participantes e possibilitar a construção teórica a partir dos dados empíricos, contribuindo para o desenvolvimento de estratégias direcionadas à promoção do bem-estar e ao aprimoramento do desempenho acadêmico^(10)^. Assim, este estudo tem como objetivo explorar as experiências de estudantes de enfermagem em relação ao estresse na prática clínica e as estratégias de enfrentamento adotadas nesse contexto.

## MÉTODO

### Tipo/Desenho do Estudo

Estudo qualitativo a partir do referencial metodológico GT, na perspectiva corbiniana, cujo processo analítico assume dinamicidade comparativa entre diferentes etapas do processo investigativo, posicionando categorias em modelo paradigmático que favorece, de forma objetiva, a compreensão sobre o fenômeno investigado^(11,12)^. Este estudo foi conduzido de acordo com as diretrizes do COREQ^(13)^, no contexto de um projeto integrado de pesquisa sobre estresse em estudantes de enfermagem durante a prática clínica.

### Local

O estudo foi realizado em uma universidade pública localizada na Região Sudeste do Brasil, envolvendo dois cursos de graduação em Enfermagem: Bacharelado em Enfermagem (oito semestres) e Bacharelado em Enfermagem com Licenciatura (dez semestres). Ambos os cursos visam à formação de enfermeiros para atuar em diferentes cenários de atenção à saúde. Todavia, além da formação de enfermeiros, o segundo curso acrescenta dois semestres e habilita o estudante para a docência na educação profissional em enfermagem. Respeitando os princípios da GT, a inclusão dos dois cursos se deu a partir da hipótese de que o contexto pode influenciar as experiências e percepções dos estudantes sobre as práticas clínicas, assim como em relação às estratégias de enfrentamento ao estresse.

### População e Critérios de Seleção

Participaram estudantes a partir do segundo ano do curso, período no qual são iniciadas as práticas clínicas em serviços públicos de saúde. Foram excluídos estudantes em licença médica. Não houve recusas, desistências ou conflitos de interesse declarados relacionados ao cenário do estudo ou aos pesquisadores envolvidos.

### Coleta de Dados

Foram realizadas entrevistas semiestruturadas entre dezembro de 2023 e janeiro de 2024. Cumpre destacar que o tempo de coleta de dados foi favorecido pela abordagem virtual e pela dinâmica de recrutamento dos participantes descritas adiante. Ademais, os pesquisadores que realizaram as entrevistas (SSS e AACA) receberam treinamento de um pesquisador sênior com experiência em pesquisa qualitativa (IRS). Assim, enquanto um pesquisador coordenava a entrevista, o outro realizava memorandos (diários de campo), observando as reações manifestadas pelos entrevistados e suas impressões como pesquisador. Esses registros foram posteriormente incorporados à análise, servindo como apoio aos pesquisadores nas etapas de codificação e rastreabilidade do processo de construção dos conceitos, bem como para a identificação da saturação teórica dos dados.

O primeiro participante foi selecionado por conveniência para a realização de uma entrevista piloto, destinada a testar e aperfeiçoar o roteiro de coleta de dados. Após essa etapa, o instrumento foi ajustado e o participante da fase piloto não foi incluído na amostra final do estudo. A partir de sua indicação, foram identificados outros potenciais participantes que atendiam aos critérios de elegibilidade estabelecidos. Os convites foram encaminhados via *WhatsApp*, e, sucessivamente, cada participante indicou novos possíveis entrevistados. Desse modo, a composição amostral ocorreu por conveniência, orientada pelos critérios de inclusão previamente definidos. Para a caracterização dos participantes, foi aplicado questionário sociodemográfico (incluindo sexo, data de nascimento, curso de graduação e anos de estudo) e, posteriormente, para a conformação do escopo analítico, foram realizadas entrevistas semiestruturadas cujas questões direcionadoras foram elaboradas a partir de uma revisão de escopo^(14)^. Dentre as questões previstas, destacam-se as que nortearam as entrevistas, sendo: 1) *Por favor, conte-me como foi a sua vivência no ambiente de prática clínica*; 2) *O que você entende por estresse*. Adicionalmente, perguntas circulares foram realizadas para explorar a profundidade do tema. As entrevistas foram realizadas por videoconferência por meio da plataforma *Zoom Workplace*, de acesso livre, gravadas em áudio, com duração média de 35 minutos. As informações fornecidas foram verificadas à luz da estrutura curricular e pedagógica dos dois cursos, com o objetivo de assegurar a confiabilidade dos dados.

Cumpre mencionar o posicionamento epistemológico dos pesquisadores em não submeterem as entrevistas à validação de conteúdo por parte dos entrevistados, pois é da natureza da GT que os dados sejam oriundos do processo investigativo, no decurso da coleta de dados. Desse modo, informações adicionais, após eventuais reflexões, poderiam conferir vieses à pesquisa.

As entrevistas foram encerradas mediante saturação teórica dos dados, ocasião observada e discutida pelos pesquisadores a partir da compreensão de que os conceitos estavam densamente desenvolvidos. Em pesquisas realizadas com a GT, esse processo é favorecido pela simultaneidade entre coleta e análise dos dados e auxiliado pelos memorandos elaborados no decurso analítico dos dados. A saturação teórica foi analisada, discutida e deliberada por dois pesquisadores (SSS e IRS).

### Análise e Tratamento dos Dados

As entrevistas foram transcritas com apoio do *MacWhisper*. Adicionalmente, para assegurar a confiabilidade, tais entrevistas foram revisadas manualmente. Seguindo os preceitos da GT, o processamento e a análise dos dados ocorreram simultaneamente à realização de cada entrevista, o que possibilitou ciclos sucessivos de codificação contínua e comparação constante entre a coleta e a análise de dados. Essa abordagem permite que os pesquisadores identifiquem o desenvolvimento de conceitos a partir da comparação constante dos dados.

Nesse sentido, cabe destacar as etapas analíticas utilizadas para o desenvolvimento de categorias/conceitos e suas respectivas subcategorias/princípios, a saber: codificações aberta, axial e integração seletiva^(11,12)^. A codificação aberta foi iniciada com a microanálise (análise linha por linha das entrevistas), etapa em que os dados foram segmentados em partes distintas mediante identificação de suas propriedades e dimensões. Por propriedades e dimensões entendem-se as particularidades das informações que apresentam nuances de qualidades, intensidades e/ou profundidades distintas, ainda que tratem de respostas às perguntas que são comuns a um determinado grupo amostral. Desse processo, resultou a identificação de códigos preliminares a partir do agrupamento de dados por similaridades entre propriedades e dimensões.

Em seguida, houve o agrupamento de códigos preliminares cujo resultado conferiu o desenvolvimento de códigos conceituais. Os códigos conceituais são elementos mais abrangentes, menos descritivos e que projetam maior densidade analítica, mas ainda de forma fragmentada. Iniciou-se, a partir desse momento, a etapa de codificação axial, mediante análise comparativa entre os códigos conceituais, momento em que os pesquisadores desenvolveram as subcategorias que são unidades explicativas mais densas sobre a realidade estudada e conformam princípios que passarão a integrar conceitos/categorias. Todavia, a natureza *grounded* do método solicita que as subcategorias/princípios orbitem uma categoria, conforme sinalizado, cuja densidade explicativa envolva todos os elementos descritos no conjunto dos códigos conceituais que constituíram as subcategorias^(11,12)^.

Na codificação integrativa, as categorias foram posicionadas em um modelo paradigmático que permitiu melhor coerência entre elas, de modo a conformar a densidade explicativa à teoria desenvolvida. São elementos do modelo paradigmático na perspectiva corbiniana: causas (fatores que induzem o acontecimento do fenômeno investigado); ações-interações (estratégias de intervenção – enfrentamento do estresse, para o contexto deste estudo); possíveis consequências esperadas (a partir das ações-interações estabelecidas)^(11,12)^.

### Aspectos Éticos

Todos os participantes assinaram o Termo de Consentimento Livre e Esclarecido previamente à coleta de dados. O estudo foi aprovado pelo Comitê de Ética em Pesquisa (CEP), sob o número de aprovação CAAE: 69618723.9.0000.5393. As entrevistas, assim como as transcrições, foram armazenadas em uma pasta restrita no *Google Drive*, com acesso limitado apenas aos pesquisadores envolvidos. Para garantir anonimato, os entrevistados, bem como seus dados, foram identificados a partir da letra “E”, referindo-se à “estudante”, seguida da respectiva ordem numérica da entrevista (E01, E02, E03…), acompanhados da categorização do curso “B” para Bacharelado e “BL” para Bacharelado e Licenciatura, além da indicação do ano de estudo.

## RESULTADOS

A partir dos critérios de elegibilidade e de saturação teórica, 15 estudantes compuseram dois grupos amostrais, sendo sete do curso de Bacharelado em Enfermagem e oito do curso de Bacharelado e Licenciatura em Enfermagem. Os estudantes apresentaram idades entre 21 e 30 anos (média de 23 anos), cursando do terceiro ao quinto ano. Nesse sentido, o constructo teórico intitulado “Transformações na prática clínica” explica fatores contextuais do estresse vivenciado durante a prática clínica e subsidia ações e intervenções voltadas às necessidades de estudantes de enfermagem (Quadro 1).

**Quadro 1:** Categorias e subcategorias – Ribeirão Preto, SP, Brasil, 2024.

Categoria	Subcategoria
3.1 Fatores contextuais do estresse na prática clínica de estudantes de enfermagem	3.1.1 Início da prática clínica: insegurança e incerteza
	3.1.2 Influência do docente na experiência da prática clínica
	3.1.3 Influência de estudantes veteranos nas expectativas sobre a prática clínica
3.2 Estratégias de enfrentamento do estresse entre estudantes de enfermagem	3.2.1 Estratégias individuais de enfrentamento do estresse
	3.2.2 Estratégias coletivas de enfrentamento do estresse

Para este artigo, dada a densidade dos resultados, são apresentadas as categorias que conformam o componente causal a partir dos fatores contextuais na prática clínica de estudantes de enfermagem, e o componente ações-interações, mediante a identificação e as estratégias de enfrentamento do estresse vivenciado por estudantes de enfermagem, no âmbito da prática clínica.

Para os estudantes, a transição do ambiente teórico para a prática clínica desencadeia uma resposta inicial de estresse. No entanto, de acordo com os participantes, quando acompanhada por estratégias de enfrentamento, favorece a adaptação; quando não, pode resultar em repercussões físicas e mentais. A Figura 1 ilustra esse processo de progressão do estresse e os caminhos de enfrentamento identificados.

**Figura 1:** Experiências e estratégias de enfrentamento de estudantes de enfermagem na prática clínica.

### Fatores Contextuais do Estresse na Prática Clínica de Estudantes de Enfermagem

Essa categoria compreende três subcategorias que expressam como o contexto influencia o início, o desenvolvimento e a vivência da prática clínica, sem uma sequência fixa, dependendo das condições de estresse e das estratégias de enfrentamento adotadas. Cumpre destacar que os trechos de depoimentos apresentados são meramente ilustrativos na representatividade das subcategorias, uma vez que resultam de uma intensa análise comparativa entre entrevistas diversas.

#### Início da Prática Clínica: Insegurança e Incerteza

As experiências iniciais na prática clínica foram marcadas por medo, insegurança e incerteza, relacionados ao receio de causar danos aos pacientes, à inexperiência e à dificuldade de tomada de decisão.

*“Eu ficava receosa mesmo da minha atuação quanto profissional – com medo mesmo de não conseguir dar a atenção necessária e o suporte necessário aos pacientes, que apesar de eu ser aluna e saber que eu estava sujeita a erros* [...] *eu sempre quero o melhor pro paciente e o melhor também para as pessoas que estão comigo na equipe, e eu acabo me cobrando muito.”* (E02, BL, 3º ano)

A transição do laboratório para o cuidado real gerou ansiedade, intensificada pela sobrecarga de tarefas, atenção simultânea a múltiplas demandas e medo de errar.

*“Acho que o acúmulo de atividades também, porque tem muita coisa para se preocupar também, tem muitas outras tarefas dentro da própria disciplina, né?* [...] *cada matéria tem sua particularidade, então são muitos detalhes que você precisa se atentar e que são cobrados também na prática.”* (E01, B, 3º ano)

A preparação prévia contribuiu para reduzir o estresse inicial.

*“Esse direcionamento nessas outras disciplinas que eu acho que deixou a gente mais seguros e mais tranquilos e diminuiu um pouco esse estresse de chegar no primeiro dia e não saber nada, de não ter esse apoio, de não ter essa rede, sabe?* (E04, B, 3º ano)

A adaptação ocorreu de forma gradual, associada ao desenvolvimento da confiança e à percepção de apoio.

*“Mas, com o passar dos dias, eu fui sentindo um pouco mais de segurança, porque acho que o grande ponto, assim, é que eu não estava sozinha, sabe?”* (E09, BL, 5º ano)

#### Influência do Docente na Experiência da Prática Clínica

Os participantes destacaram a influência do docente como determinante para a vivência da prática clínica. Posturas que favoreciam autonomia, participação e *feedback* construtivo estiveram associadas a experiências positivas.

*“Teve alguns estágios de prática clínica que eu gostei bastante, por conta de como o professor deixou a gente trabalhar no estágio, e teve outros que eu realmente não gostei. Acho que isso influenciou diretamente na experiência.”* (E01, B, 3º ano)

“*Eu entendo que precisa ter um acompanhamento, mas tinha docente que dava muito mais liberdade e abertura para a gente fazer as coisas, procedimentos etc. E teve outros que não tinham confiança e pegavam para fazer, sabe? Não davam oportunidade do aluno fazer, preferia fazer e o aluno observar.”* (E01, B, 3º ano)

De acordo com os estudantes, o apoio docente reduziu o medo e fortaleceu a confiança.

*“E eu sinto que ela me deixou muito mais à vontade, preparada, mais segura para fazer a parte desse procedimento. E tirou um pouquinho aquele medo de “eu vou matar o paciente, eu ainda sou estudante e não vou fazer nada”.* [...] *Eu me senti muito segura para ir avançando na parte mais prática mesmo.”* (E15, B, 3º ano)

Ambientes acolhedores favoreceram a segurança emocional, enquanto posturas autoritárias intensificaram a ansiedade.

*“Quando os professores são gentis e compreendem nosso contexto que somos estudantes, ainda aprendendo e desenvolvendo nossas habilidades, nos sentimos muito mais livres e aprendemos muito mais.”* (E08, B, 3º ano)

#### Influência de Estudantes Veteranos nas Expectativas Sobre a Prática Clínica

Os dados sinalizaram que os relatos de estudantes veteranos influenciaram expectativas antes do início da prática, especialmente quando associados a experiências negativas.

*“Acho que aqui, na escola, existe muito preconceito em relação à disciplina. Ouvimos de estudantes veteranos* [...] *que é uma disciplina difícil, que não somos bem recebidos e que temos que aguentar muita coisa”.* (E04, B, 3º ano)

*“Eu estava com muito medo para começar a clínica, ainda mais que é uma disciplina que o pessoal fala bastante*”. (E11, B, 3º ano)

*No início, eu fiquei um pouco mais receosa, porque muita fala de veterano falandoque as professoras são, assim, um pouco chatas em relação aos estágios e,realmente, são muito rígidas. Só que eu tive uma experiência excelente, assim, nãotive nenhuma experiência ruim com as professoras.* (E02, BL, 3º ano)

Apesar disso, alguns participantes relataram experiências positivas que superaram expectativas iniciais.

[...] *Foi bem mais tranquilo do que falavam*. (E15, B, 3º ano)

### Estratégias de Enfrentamento do Estresse entre Estudantes de Enfermagem

Os estudantes identificaram estratégias individuais e coletivas para lidar com o estresse decorrente da prática clínica, além de sugerirem ações institucionais de apoio, conforme destacam as subcategorias adiante.

#### Estratégias Individuais de Enfrentamento do Estresse

Atividades físicas, práticas de relaxamento e redes de apoio foram descritas como fundamentais para o manejo do estresse.

*“Acho que algo muito importante é conversar com nossa rede de apoio, sabe? Amigos ou professores. E, a partir disso, identificar aspectos-chave que nos ajudem a lidar com a situação e enfrentar o estresse da melhor forma possível.”* (E06, BL, 5º ano)

O apoio de docentes e profissionais de saúde favoreceu a segurança e pertencimento.

*“Poder conversar, de pessoa para pessoa, sem aquela sensação de hierarquia, sabe? Tipo professor e aluno. Poder expressar como você se sentiu em determinado momento e ter alguém que te ajude dentro do que é possível.”* (E07, B, 3º ano)

O acompanhamento psicológico também foi reconhecido como recurso relevante para o manejo do estresse.

#### Estratégias Coletivas de Enfrentamento do Estresse

Rodas de conversa e espaços coletivos foram considerados importantes para o compartilhamento de experiências, fortalecimento de vínculos e redução do estresse.

*“Criar um ambiente saudável, um ambiente de comunidade mesmo sustentável, é muito importante para que esses alunos se sintam à vontade e consigam, assim, em situações que não precisam de estresse, eles não se sintam estressados*.” (E05, BL, 3º ano)

Os participantes destacaram a importância de envolver gestores, docentes e profissionais em espaços de diálogo.

*“Eu acho que caberia à equipe gestora ali conseguir alguma roda de conversa com os profissionais que pudessem, de fato, entender mesmo aquilo, o motivo do que está causando grande estresse, assim, no pessoal, na equipe.”* (E02, BL, 3º ano)

*“Eu penso que juntar a equipe para conversar sobre as potencialidades ou as fragilidades do serviço e das práticas de cada um ali também, isso acaba potencializando a prática clínica de todo mundo*.” (E09, BL, 5º ano)

Foram sugeridas estratégias institucionais, como ajuste da carga horária, integração progressiva à prática, protocolos de manejo de crises, tutoria individual e coletiva, além de simulações e *debriefings.*

*“Eu acho que seria legal se a gente tivesse na graduação algo, não sei, algum grupo ou alguma disciplina mesmo que ajudasse, assim, a ter uns bate-papos em relação a como enfrentar o estresse, sabe?”* (E13, BL, 5º ano)

A experiência clínica mostrou-se afetada pelo ambiente, pelas relações interpessoais e pelas atitudes docentes, articuladas às expectativas e experiências prévias.

## DISCUSSÃO

A prática clínica é um componente essencial da formação em enfermagem, permitindo a aplicação do conhecimento teórico, o desenvolvimento de habilidades e a compreensão das dinâmicas do trabalho profissional^(6,15)^. Esse momento é marcado por apreensão, estresse e necessidade de adaptação a um ambiente desconhecido e exigente. Tais experiências, comuns diante do desconhecido, tendem a diminuir com o processo de adaptação. Contudo, quando não adequadamente manejado, o estresse pode atingir níveis prejudiciais^(16)^.

A familiarização prévia com o cenário clínico mostra-se fundamental, uma vez que o ambiente influencia a construção da identidade profissional e está associado a níveis moderados de estresse durante os estágios^(15,17)^. As expectativas dos estudantes moldadas por relatos de colegas veteranos e pela atuação dos educadores influenciam diretamente a percepção do estresse e o processo de adaptação, reforçando a importância de intervenções educativas que promovam expectativas realistas, de modo a promover a ruptura de estigmas que possam potencializar a ansiedade e, por conseguinte, o estresse nas práticas clínicas^(18)^. O papel do educador emerge como central na experiência da prática clínica, uma vez que estratégias pedagógicas, formas de interação e níveis de apoio impactam diretamente a satisfação, a aprendizagem e o engajamento dos estudantes^(8,9,19)^. Abordagens colaborativas e acolhedoras favorecem autonomia, segurança emocional e senso de pertencimento, enquanto posturas excessivamente controladoras podem limitar a participação e intensificar a insegurança^(9)^.

Estudos mostram que experiências positivas e maior satisfação estão associadas a estratégias eficazes de enfrentamento do estresse, enquanto o ano de formação também influencia esse processo: estudantes mais avançados tendem a adotar a resolução de problemas de forma mais eficiente^(20,21)^. Nesse contexto, os resultados deste estudo indicam que os estudantes recorreram a estratégias individuais e coletivas para lidar com os estressores. Entre as individuais, destacam-se reestruturação cognitiva, apoio social, práticas de bem-estar (exercício, respiração, música, leitura), acompanhamento psicológico e foco nas tarefas.

Por outro lado, estilos passivos, como evitar, postergar ou enfrentar problemas isoladamente, relacionam-se a maior estresse e apenas alívio temporário, podendo comprometer o manejo de situações críticas^(20)^. Em contraste, atitudes otimistas favorecem menor estresse e uso de estratégias de resolução de problemas, geralmente apoiadas por colegas, familiares e educadores^(15)^. Além disso, práticas de bem-estar, como exercício físico, embora classificadas por alguns estudos como estratégias de transferência de eficácia limitada, mostram associação com inteligência emocional, autoeficácia e regulação emocional^(22)^. Considerando que a autoeficácia contribui para melhor manejo do estresse e desempenho clínico^(20)^, tais práticas podem favorecer o equilíbrio emocional, ainda que sejam necessários novos estudos para confirmar sua efetividade. O suporte social destacou-se como fator protetor, especialmente quando mediado pela participação ativa de educadores^(23,24)^. Estratégias coletivas, como grupos de discussão, *debriefings* e espaços institucionais de diálogo, foram apontadas como desejáveis para o compartilhamento de experiências, fortalecimento de vínculos e redução do estresse.

O manejo do estresse começa com a preparação dos estudantes para a prática clínica, por meio de medidas que reduzem ansiedade e desenvolvem habilidades. Cursos que abordem causas e efeitos do estresse e fortaleçam autoconfiança, autoeficácia e resiliência são fundamentais para enfrentar desafios clínicos^(20,25)^. Nesse sentido, os resultados reforçam a necessidade de iniciativas estruturadas para preparação clínica, manejo do estresse e promoção do bem-estar, incluindo programas educativos, tutoriais, simulações realistas e atividades de regulação emocional^(15,26,27,28,29)^. Programas baseados em *mindfulness* e redes de apoio social também demonstram benefícios relevantes^(30)^. No âmbito organizacional, esforços conjuntos entre instituições de ensino, serviços de saúde, educadores e preceptores são fundamentais para criar ambientes de prática acolhedores e comprometidos com a formação em enfermagem, reduzindo estressores e favorecendo a aprendizagem^(8)^. O uso de *debriefings* ao final das atividades práticas destaca-se como estratégia relevante para avaliação, *feedback* e aprimoramento do processo formativo.

Em conjunto, os achados deste estudo indicam que o estresse é vivenciado individualmente pelos estudantes, mas produzido, intensificado ou atenuado por fatores coletivos, relacionais e institucionais, configurando-se como um fenômeno coproduzido no interior do contexto formativo. O estresse inicial não emerge apenas “de dentro” do estudante, mas se constrói a partir de condições concretas da prática clínica, como o início abrupto das atividades, a carga de tarefas e a forma como o estágio é organizado, articuladas às relações interpessoais estabelecidas nos cenários de aprendizagem, especialmente à atuação do educador e aos significados previamente atribuídos à prática por meio de relatos de estudantes veteranos.

Além disso, a dimensão coletiva da formação atravessa a maneira como o estresse é percebido e manejado, considerando a presença ou ausência de espaços institucionais de escuta, o apoio entre pares e as possibilidades de compartilhamento de experiências. Essa compreensão amplia a leitura do estresse na prática clínica como um processo dinâmico, influenciado pelas condições formativas, e contribui para refletir sobre como diferentes arranjos pedagógicos e institucionais podem repercutir na adaptação dos estudantes ao longo da formação em enfermagem.

## CONCLUSÃO

Este estudo permitiu compreender que a prática clínica é vivenciada por estudantes de enfermagem como um momento formativo marcado por insegurança, desconforto e estresse, especialmente no início das atividades, quando há menor familiaridade com o ambiente, as demandas do cuidado e as relações estabelecidas nos cenários de prática. Ao explorar as experiências e percepções dos estudantes, os resultados evidenciam que esse estresse não se manifesta de forma homogênea, mas é moldado pelas condições pedagógicas, relacionais e institucionais em que a prática clínica se desenvolve.

A identificação de estratégias individuais e coletivas de enfrentamento amplia a compreensão sobre como os estudantes lidam com os desafios da prática clínica e evidencia que o manejo do estresse pode favorecer processos de adaptação mais seguros ao longo da formação. Nesse sentido, o estudo contribui ao demonstrar que o estresse percebido não constitui um obstáculo fixo ou inevitável, mas uma experiência passível de reconhecimento e manejo por meio de ações educativas, suporte social e organização institucional mais sensível às necessidades dos estudantes.

Ao integrar essas dimensões, os achados oferecem subsídios para o aprimoramento de práticas pedagógicas e institucionais que qualifiquem a experiência clínica, favoreçam a permanência e o desenvolvimento dos estudantes e sustentem seu percurso formativo ao longo das diferentes etapas da formação em enfermagem. Além disso, os achados podem orientar pesquisas futuras, especialmente estudos longitudinais que avaliem o impacto de intervenções educacionais sobre o estresse ao longo da formação em enfermagem.

## Data Availability

Todo o conjunto de dados que dá suporte aos resultados deste estudo foi publicado no próprio artigo.

# Translation: El estrés y las estrategias de enfrentamiento entre los estudiantes de enfermería en prácticas clínicas: un estudio cualitativo*

**DOI:** 10.1590/1980-220X-REEUSP-2026-0056es

**Authors:** Sara Soares dos Santos, Ítalo Rodolfo Silva, Agostinho A. C. Araújo, Simone de Godoy, Emerson Willian Santos de Almeida, Lucas Gardim, Kristina Mikkonen, Isabel Amélia Costa Mendes

**Affiliations:** 1 Universidade de São Paulo, Escola de Enfermagem de Ribeirão Preto, Ribeirão Preto, SP, Brasil.; 2 Universidade Federal do Rio de Janeiro, Escola de Enfermagem Anna Nery, Rio de Janeiro, RJ, Brasil.; 3 University of Oulu, Faculty of Medicine, Research Unit of Health Sciences and Technology, Oulu, Finland.

**Keywords:** Estrés Psicológico, Educación en Enfermería, Estudiantes de Enfermería

## Abstract

**Objetivo:**

Analizar las experiencias de los estudiantes de enfermería en relación con el estrés en la práctica clínica y las estrategias de afrontamiento adoptadas en ese contexto.

**Método:**

Estudio cualitativo, cuyo marco metodológico fue el *Grounded Theory* (GT), desde la perspectiva de Corbin, realizado con 15 estudiantes de grado en enfermería de una universidad pública de la región sudeste de Brasil.

**Resultados:**

Para los estudiantes de enfermería, el inicio de las prácticas clínicas estuvo marcado por el estrés, la inseguridad y las dificultades de adaptación, factores influidos por la organización de las actividades, las relaciones establecidas en los entornos de prácticas y el apoyo disponible. Las estrategias de afrontamiento incluyeron: 1) acciones individuales, como la regulación emocional y la búsqueda de apoyo; y 2) acciones colectivas, como el apoyo entre compañeros y los espacios institucionales de diálogo.

**Conclusión:**

El estrés en la práctica clínica se presenta como una experiencia formativa desafiante, en la que influyen factores individuales, relacionales e institucionales. Reconocer estas dimensiones amplía las posibilidades de intervención pedagógica e institucional, lo que contribuye a mejorar la calidad de la formación en el contexto de la práctica clínica y a la adaptación de los estudiantes a lo largo de su formación en enfermería.

## INTRODUCCIÓN

Los estudiantes constituyen un grupo particularmente vulnerable a las experiencias estresantes, y el estrés académico se reconoce como un desafío relevante de salud pública debido a sus impactos en la salud mental^(1)^. En el ámbito de la formación en salud, los estudiantes de enfermería presentan niveles más elevados de estrés en comparación con los de otras áreas, sobre todo debido al contacto prolongado con los pacientes durante la práctica clínica^(2)^. Aunque la práctica clínica es fundamental para vincular el conocimiento teórico con la aplicación práctica^(3)^, la evidencia indica que este entorno suele percibirse como desafiante y generador de estrés^(3,4)^.

Entre los principales factores estresantes identificados en este contexto, se destacan la inseguridad respecto a la formación teórica y práctica, el elevado sentido de responsabilidad ante el cuidado del paciente, la percepción de insuficiencia de habilidades, la presión del tiempo y las dificultades en las relaciones interpersonales^(5)^. Dichos factores pueden afectar la calidad de vida de los estudiantes, con repercusiones negativas en su vida personal y su desempeño académico^(4)^.

A pesar de las continuas transformaciones en los sistemas de salud y de la creciente complejidad de los entornos clínicos, persiste una comprensión limitada sobre la organización y estructuración de la exposición de los estudiantes de enfermería a la práctica clínica. La educación clínica aún privilegia los enfoques a corto plazo, orientados a tareas, en detrimento de la preparación para los desafíos futuros y los cambios en la práctica profesional^(6)^. Aunque estudios previos han buscado explorar estas cuestiones^(7,8,9)^, muchos no abordan en profundidad las experiencias subjetivas de los estudiantes. Ante esto, este estudio busca responder a las siguientes preguntas: ¿cuáles son las experiencias y percepciones de los estudiantes de enfermería respecto al entorno de la práctica clínica y qué estrategias de enfrentamiento adoptan para lidiar con los desafíos en este contexto?

En este contexto, la Teoría fundamentada (llamada en inglés *Grounded Theory – GT*) constituye un enfoque adecuado para investigar estas cuestiones, ya que valora las experiencias de los participantes y permite la construcción teórica a partir de los datos empíricos, contribuyendo al desarrollo de estrategias orientadas a promover el bienestar y mejorar el desempeño académico^(10)^. Por lo tanto, este estudio tiene como objetivo explorar las experiencias de los estudiantes de enfermería en relación con el estrés en la práctica clínica y las estrategias de enfrentamiento adoptadas en ese contexto.

## MÉTODO

### Tipo/Diseño del Estudio

Estudio cualitativo basado en el marco metodológico de la GT, desde la perspectiva corbiniana, cuyo proceso analítico asume una dinámica comparativa entre las diferentes etapas del proceso de investigación, situando las categorías en un modelo paradigmático que favorece, de manera objetiva, la comprensión del fenómeno investigado^(11,12)^. Este estudio se llevó a cabo de acuerdo con las directrices del COREQ^(13)^, en el marco de un proyecto integrado de investigación sobre el estrés en estudiantes de enfermería durante la práctica clínica.

### Lugar

El estudio se llevó a cabo en una universidad pública ubicada en la región sudeste de Brasil, y abarcó dos programas de pregrado en enfermería: la Licenciatura en Enfermería (ocho semestres) y la Licenciatura en Enfermería con Certificación Docente (diez semestres). Ambos programas tienen como objetivo formar enfermeros para que ejerzan en diferentes entornos de atención en salud. Sin embargo, además de la formación de enfermeros, el segundo programa añade dos semestres y capacita al estudiante para la docencia en la educación profesional en enfermería. Respetando los principios de la GT, la inclusión de los dos programas se basó en la hipótesis de que el contexto puede influir en las experiencias y percepciones de los estudiantes sobre las prácticas clínicas, así como en las estrategias para afrontar el estrés.

### Población y Criterios de Selección

Participaron estudiantes a partir del segundo año del programa, período en el que se inician las prácticas clínicas en servicios públicos de salud. Se excluyó a los estudiantes con licencia médica. No hubo rechazos, desistimientos ni conflictos de interés declarados relacionados con el contexto del estudio o con los investigadores involucrados.

### Recolección de Datos

Se realizaron entrevistas semiestructuradas entre diciembre de 2023 y enero de 2024. Cabe destacar que el tiempo de recolección de datos se vio favorecido por el enfoque virtual y por la dinámica de reclutamiento de los participantes que se describe a continuación. Además, los investigadores que realizaron las entrevistas (SSS y AACA) recibieron capacitación de un investigador senior con experiencia en investigación cualitativa (IRS). Así, mientras un investigador coordinaba la entrevista, el otro elaboraba notas (diarios de campo), observando las reacciones manifestadas por los entrevistados y sus impresiones como investigador. Estos registros se incorporaron posteriormente al análisis, sirviendo de apoyo a los investigadores en las etapas de codificación y trazabilidad del proceso de construcción de los conceptos, así como para la identificación de la saturación teórica de los datos.

El primer participante fue seleccionado por conveniencia para realizar una entrevista piloto, destinada a probar y perfeccionar el guion de Recolección de datos. Tras esta etapa, se ajustó el instrumento y el participante de la fase piloto no fue incluido en la muestra final del estudio. A partir de su recomendación, se identificaron otros posibles participantes que cumplían con los criterios de elegibilidad establecidos. Las invitaciones se enviaron a través de *WhatsApp* y, sucesivamente, cada participante recomendó a nuevos posibles entrevistados. De esta manera, la composición de la muestra se realizó por conveniencia, guiada por los criterios de inclusión previamente definidos. Para la caracterización de los participantes, se aplicó un cuestionario sociodemográfico (que incluía sexo, fecha de nacimiento, carrera universitaria y años de estudio) y, posteriormente, para definir el alcance analítico, se realizaron entrevistas semiestructuradas cuyas preguntas orientativas se elaboraron a partir de una revisión bibliográfica^(14)^. Entre las preguntas previstas, se destacan aquellas que orientaron las entrevistas, a saber: 1) *Por favor, cuénteme cómo fue su experiencia en el entorno de práctica clínica*; 2) *¿Qué entiende usted por estrés*? Además, se formularon preguntas abiertas para explorar el tema en profundidad. Las entrevistas se realizaron por videoconferencia a través de la plataforma *Zoom Workplace*, de acceso libre, se grabaron en audio y tuvieron una duración promedio de 35 minutos. La información proporcionada se verificó a la luz de la estructura curricular y pedagógica de ambos cursos, con el objetivo de garantizar la confiabilidad de los datos.

Cabe mencionar la postura epistemológica de los investigadores de no someter las entrevistas a la validación de contenido por parte de los entrevistados, ya que es propio de la GT que los datos surjan del proceso de investigación, durante la Recolección de datos. De este modo, la información adicional, tras posibles reflexiones, podría introducir sesgos en la investigación.

Las entrevistas se dieron por concluidas al alcanzarse la saturación teórica de los datos, momento observado y discutido por los investigadores a partir de la comprensión de que los conceptos se habían desarrollado en profundidad. En las investigaciones realizadas con la GT, este proceso se ve favorecido por la simultaneidad entre la Recolección y el análisis de los datos, y se ve respaldado por los memorandos elaborados durante el proceso analítico de los datos. La saturación teórica fue analizada, discutida y deliberada por dos investigadores (SSS e IRS).

### Análisis y Procesamiento de los Datos

Las entrevistas se transcribieron con la ayuda de *MacWhisper*. Además, para garantizar la confiabilidad, dichas entrevistas se revisaron manualmente. Siguiendo los principios de la GT, el procesamiento y el análisis de los datos se realizaron simultáneamente a la realización de cada entrevista, lo que permitió ciclos sucesivos de codificación continua y comparación constante entre la Recolección y el análisis de los datos. Este enfoque permite a los investigadores identificar el desarrollo de conceptos a partir de la comparación constante de los datos.

En este sentido, cabe destacar las etapas analíticas utilizadas para el desarrollo de categorías/conceptos y sus respectivas subcategorías/principios, a saber: codificación abierta, axial e integración selectiva^(11,12)^. La codificación abierta se inició con el microanálisis (análisis línea por línea de las entrevistas), etapa en la que los datos se segmentaron en partes distintas mediante la identificación de sus propiedades y dimensiones. Por propiedades y dimensiones se entienden las particularidades de la información que presentan matices de cualidades, intensidades y/o profundidades distintas, aunque se trate de respuestas a preguntas que son comunes a un determinado grupo de la muestra. De este proceso resultó la identificación de códigos preliminares a partir de la agrupación de datos por similitudes entre propiedades y dimensiones.

A continuación, se agruparon los códigos preliminares, lo que dio lugar al desarrollo de códigos conceptuales. Los códigos conceptuales son elementos más amplios, menos descriptivos y que presentan una mayor densidad analítica, aunque aún de manera fragmentada.

A partir de ese momento, se inició la etapa de codificación axial, mediante un análisis comparativo entre los códigos conceptuales, momento en el que los investigadores desarrollaron las subcategorías, que son unidades explicativas más densas sobre la realidad estudiada y conforman principios que pasarán a integrar conceptos/categorías. Sin embargo, la naturaleza *grounded* del método exige que las subcategorías/principios giren en torno a una categoría, tal como se ha señalado, cuya densidad explicativa abarque todos los elementos descritos en el conjunto de los códigos conceptuales que constituyeron las subcategorías^(11,12)^.

En la codificación integradora, las categorías se ubicaron en un modelo paradigmático que permitió una mayor coherencia entre ellas, a fin de ajustar la densidad explicativa a la teoría desarrollada. Los elementos del modelo paradigmático desde la perspectiva de Corbin son: causas (factores que provocan la ocurrencia del fenómeno investigado); acciones-interacciones (estrategias de intervención —manejo del estrés, en el contexto de este estudio—); posibles consecuencias esperadas (a partir de las acciones-interacciones establecidas)^(11,12)^.

### Aspectos Éticos

Todos los participantes firmaron el Formulario de Consentimiento Libre e Informado antes de la Recolección de datos. El estudio fue aprobado por el Comité de Ética en Investigación (CEP), con el número de aprobación CAAE: 69618723.9.0000.5393. Las entrevistas, así como las transcripciones, se almacenaron en una carpeta restringida en *Google Drive*, con acceso limitado únicamente a los investigadores involucrados. Para garantizar el anonimato, las entrevistadas, así como sus datos, se identificaron con la letra “E”, que hace referencia a “estudiante”, seguida del orden numérico correspondiente a la entrevista (E01, E02, E03…), junto con la categorización del programa de estudios “B” para la licenciatura y “BL” para la licenciatura y el magisterio, además de la indicación del año de estudio.

## RESULTADOS

A partir de los criterios de elegibilidad y de saturación teórica, 15 estudiantes formaron dos grupos de muestra, siete de ellos del programa de Licenciatura en Enfermería y ocho del programa de Licenciatura y Maestría en Enfermería. Los estudiantes tenían entre 21 y 30 años (promedio de 23 años) y cursaban desde el tercer hasta el quinto año. En este sentido, el constructo teórico titulado “Transformaciones en la práctica clínica” explica los factores contextuales del estrés experimentado durante la práctica clínica y respalda acciones e intervenciones orientadas a las necesidades de los estudiantes de enfermería (Cuadro 1).

**Cuadro 1:** Categorías y subcategorías – Ribeirão Preto, SP, Brasil, 2024.

Categoría	Subcategoría
3.1 Factores contextuales del estrés en la práctica clínica de los estudiantes de enfermería	3.1.1 Inicio de la práctica clínica: inseguridad e incertidumbre
	3.1.2 Influencia del docente en la experiencia de la práctica clínica
	3.1.3 Influencia de los estudiantes de cursos superiores en las expectativas sobre la práctica clínica
3.2 Estrategias de enfrentamiento del estrés entre los estudiantes de enfermería	3.2.1 Estrategias individuales para enfrentar el estrés
	3.2.2 Estrategias colectivas para enfrentar el estrés

Para este artículo, dada la densidad de los resultados, se presentan las categorías que conforman el componente causal a partir de los factores contextuales en la práctica clínica de los estudiantes de enfermería, y el componente de acciones e interacciones, mediante la identificación y las estrategias de enfrentamiento del estrés experimentado por los estudiantes de enfermería, en el ámbito de la práctica clínica.

Para los estudiantes, la transición del entorno teórico a la práctica clínica desencadena una respuesta inicial de estrés. Sin embargo, según los participantes, cuando va acompañada de estrategias de enfrentamiento, favorece la adaptación; de lo contrario, puede tener repercusiones físicas y mentales. La Figura 1 ilustra este proceso de progresión del estrés y las vías de enfrentamiento identificadas.

**Figura 1:** Experiencias y estrategias de enfrentamiento de los estudiantes de enfermería en la práctica clínica.

### Factores Contextuales del Estrés en la Práctica Clínica de los Estudiantes de Enfermería

Esta categoría comprende tres subcategorías que expresan cómo el contexto influye en el inicio, el desarrollo y la experiencia de la práctica clínica, sin una secuencia fija, dependiendo de las condiciones de estrés y de las estrategias de enfrentamiento adoptadas. Cabe destacar que los fragmentos de los testimonios presentados son meramente ilustrativos de la representatividad de las subcategorías, ya que son el resultado de un intenso análisis comparativo entre diversas entrevistas.

#### Inicio de la Práctica Clínica: Inseguridad e Incertidumbre

Las experiencias iniciales en la práctica clínica estuvieron marcadas por el miedo, la inseguridad y la incertidumbre, relacionados con el temor a causar daño a los pacientes, la inexperiencia y la dificultad para tomar decisiones.

*“Realmente me sentía insegura respecto a mi desempeño como profesional; tenía miedo de no poder brindar la atención y el apoyo necesarios a los pacientes, y aunque era estudiante y sabía que estaba sujeta a cometer errores* [...]*siempre quiero lo mejor para el paciente y también lo mejor para las personas que están conmigo en el equipo, y termino exigiéndome mucho.”* (E02, BL, tercer año)

La transición del laboratorio a la atención real generó ansiedad, intensificada por la sobrecarga de tareas, la atención simultánea a múltiples exigencias y el miedo a equivocarse.

*“Creo que también la acumulación de actividades, porque hay muchas cosas de las que preocuparse, hay muchas otras tareas dentro de la propia disciplina, ¿no?* [...] *cada materia tiene sus particularidades, así que hay muchos detalles a los que hay que prestar atención y que también se exigen en la práctica.”* (E01, B, tercer año)

La preparación previa ayudó a reducir el estrés inicial.

*“Esa orientación en esas otras disciplinas creo que nos hizo sentir más seguros y tranquilos, y disminuyó un poco ese estrés de llegar el primer día y no saber nada, de no tener ese apoyo, de no tener esa red, ¿sabes?* (E04, B, tercer año)

La adaptación se dio de manera gradual, junto con el desarrollo de la confianza y la percepción de apoyo.

*“Pero, a medida que pasaban los días, fui sintiéndome un poco más segura, porque creo que lo más importante es que no estaba sola, ¿sabes?”* (E09, BL, 5.º año)

#### Influencia del Docente en la Experiencia de la Práctica Clínica

Los participantes destacaron la influencia del docente como un factor determinante para la vivencia de la práctica clínica. Las actitudes que favorecían la autonomía, la participación y el feedback constructivo se asociaron con experiencias positivas.

*“Hubo algunas prácticas clínicas que me gustaron mucho, por la forma en que el profesor nos dejó trabajar durante la práctica, y hubo otras que realmente no me gustaron. Creo que eso influyó directamente en la experiencia.”* (E01, B, tercer año)

*“Entiendo que se necesita un acompañamiento, pero había profesores que nos daban mucha más libertad y apertura para hacer las cosas, los procedimientos, etc.*

*Y hubo otros que no confiaban en nosotros y se encargaban de hacerlo ellos mismos, ¿sabes? No le daban la oportunidad al estudiante de hacerlo, preferían hacerlo ellos y que el estudiante observara.”* (E01, B, tercer año)

Según los estudiantes, el apoyo de los docentes redujo el miedo y fortaleció la confianza.

*“Y siento que ella me hizo sentir mucho más a gusto, preparada y segura para realizar esa parte del procedimiento. Y me quitó un poco ese miedo de ‘voy a matar al paciente, todavía soy estudiante y no voy a poder hacer nada’.”* [...] *Me sentí muy segura para seguir avanzando en la parte más práctica.”* (E15, B, tercer año)

Los ambientes acogedores favorecieron la seguridad emocional, mientras que las actitudes autoritarias intensificaron la ansiedad.

*“Cuando los profesores son amables y comprenden nuestro contexto —que somos estudiantes, que aún estamos aprendiendo y desarrollando nuestras habilidades—, nos sentimos mucho más libres y aprendemos mucho más.”* (E08, B, tercer año)

#### Influencia de los Estudiantes de Cursos Superiores en las Expectativas Sobre la Práctica Clínica

Los datos indicaron que los relatos de los estudiantes de cursos superiores influyeron en las expectativas antes del inicio de la práctica, especialmente cuando se asociaban con experiencias negativas.

*“Creo que aquí, en la escuela, hay mucho prejuicio con respecto a la disciplina. Escuchamos de los estudiantes de cursos superiores* [...] *que es una asignatura difícil, que no nos reciben bien y que tenemos que aguantar muchas cosas”.* (E04, B, tercer año)

“*Tenía mucho miedo de empezar la práctica clínica, sobre todo porque es una asignatura de la que la gente habla mucho*”. (E11, B, tercer año)

*Al principio, me sentí un poco más nerviosa, porque se oye mucho a los estudiantes de cursos superiores decir que las profesoras son, digamos, un poco pesadas con respecto a las prácticas y, de hecho, son muy estrictas. Pero tuve una experiencia excelente, así que no tuve ninguna experiencia mala con las profesoras.* (E02, BL, tercer año)

A pesar de ello, algunos participantes relataron experiencias positivas que superaron sus expectativas iniciales.

[...] *Fue mucho más tranquilo de lo que decían*. (E15, B, tercer año)

### Estrategias para Enfrentar el Estrés entre los Estudiantes de Enfermería

Los estudiantes identificaron estrategias individuales y colectivas para lidiar con el estrés derivado de la práctica clínica, además de sugerir medidas institucionales de apoyo, tal como lo destacan las subcategorías a continuación.

#### Estrategias Individuales para Enfrentar el Estrés

Las actividades físicas, las prácticas de relajación y las redes de apoyo se describieron como fundamentales para el manejo del estrés.

*“Creo que algo muy importante es hablar con nuestra red de apoyo, ¿sabes? Amigos o profesores. Y, a partir de ahí, identificar aspectos clave que nos ayuden a lidiar con la situación y enfrentar el estrés de la mejor manera posible.”* (E06, BL, 5.º año)

El apoyo de los docentes y los profesionales de la salud favoreció la seguridad y el sentido de pertenencia.

*“Poder platicar, de persona a persona, sin esa sensación de jerarquía, ¿sabes? Como de profesor y alumno. Poder expresar cómo te sentiste en un momento determinado y tener a alguien que te ayude dentro de lo posible.”* (E07, B, tercer año)

El acompañamiento psicológico también se reconoció como un recurso relevante para el manejo del estrés.

#### Estrategias Colectivas para Enfrentar el Estrés

Las rondas de conversación y los espacios colectivos se consideraron importantes para compartir experiencias, fortalecer los vínculos y reducir el estrés.

*“Crear un ambiente saludable, un ambiente comunitario verdaderamente sostenible, es muy importante para que estos estudiantes se sientan a gusto y así, en situaciones que no requieren estrés, no se sientan estresados*.” (E05, BL, tercer año)

Los participantes destacaron la importancia de involucrar a los administradores, docentes y profesionales en espacios de diálogo.

*“Creo que le correspondería al equipo directivo organizar alguna ronda de conversación con los profesionales que realmente pudieran entender eso, la razón de lo que está causando tanto estrés, tanto a nivel personal como en el equipo.”* (E02, BL, tercer año)

*“Creo que reunir al equipo para hablar sobre las fortalezas o las debilidades del servicio y de las prácticas de cada uno también termina potenciando la práctica clínica de todos*”. (E09, BL, 5.º año)

Se sugirieron estrategias institucionales, como el ajuste de la carga horaria, la integración progresiva a la práctica, protocolos de manejo de crisis, tutoría individual y colectiva, además de simulaciones y *debriefings.*

*“Creo que sería bueno que en la carrera tuviéramos algo, no sé, algún grupo o alguna asignatura que nos ayudara a tener pláticas sobre cómo lidiar con el estrés, ¿sabes?”* (E13, BL, 5.º año)

La experiencia clínica se vio afectada por el entorno, las relaciones interpersonales y las actitudes de los docentes, en relación con las expectativas y experiencias previas.

## DISCUSIÓN

La práctica clínica es un componente esencial de la formación en enfermería, ya que permite la aplicación de los conocimientos teóricos, el desarrollo de habilidades y la comprensión de las dinámicas del trabajo profesional^(6,15)^. Este momento se caracteriza por la aprensión, el estrés y la necesidad de adaptarse a un entorno desconocido y exigente. Estas experiencias, comunes ante lo desconocido, tienden a disminuir con el proceso de adaptación. Sin embargo, cuando no se maneja adecuadamente, el estrés puede alcanzar niveles perjudiciales^(16)^.

La familiarización previa con el entorno clínico resulta fundamental, ya que este influye en la construcción de la identidad profesional y se asocia con niveles moderados de estrés durante las prácticas^(15,17)^. Las expectativas de los estudiantes, moldeadas por los relatos de compañeros con más experiencia y por la actuación de los educadores, influyen directamente en la percepción del estrés y en el proceso de adaptación, lo que refuerza la importancia de las intervenciones educativas que promuevan expectativas realistas, con el fin de romper los estigmas que puedan potenciar la ansiedad y, por consiguiente, el estrés en las prácticas clínicas^(18)^. El papel del educador se destaca como fundamental en la experiencia de la práctica clínica, ya que las estrategias pedagógicas, las formas de interacción y los niveles de apoyo influyen directamente en la satisfacción, el aprendizaje y el compromiso de los estudiantes^(8,9,19)^. Los enfoques colaborativos y acogedores favorecen la autonomía, la seguridad emocional y el sentido de pertenencia, mientras que las actitudes excesivamente controladoras pueden limitar la participación e intensificar la inseguridad^(9)^.

Los estudios muestran que las experiencias positivas y una mayor satisfacción están asociadas a estrategias eficaces para afrontar el estrés, mientras que el año de formación también influye en este proceso: los estudiantes en cursos más avanzados tienden a resolver problemas de manera más eficiente^(20,21)^. En este contexto, los resultados de este estudio indican que los estudiantes recurrieron a estrategias individuales y colectivas para lidiar con los factores estresantes. Entre las estrategias individuales, se destacan la reestructuración cognitiva, el apoyo social, las prácticas de bienestar (ejercicio, respiración, música, lectura), el acompañamiento psicológico y el enfoque en las tareas.

Por otro lado, los estilos pasivos, como evitar, posponer o enfrentar los problemas de manera aislada, se relacionan con un mayor estrés y solo un alivio temporal, lo que puede comprometer el manejo de situaciones críticas^(20)^. En contraste, las actitudes optimistas favorecen un menor estrés y el uso de estrategias de resolución de problemas, generalmente con el apoyo de compañeros, familiares y educadores^(15)^. Además, las prácticas de bienestar, como el ejercicio físico, aunque clasificadas por algunos estudios como estrategias de transferencia de eficacia limitada, muestran una asociación con la inteligencia emocional, la autoeficacia y la regulación emocional^(22)^. Considerando que la autoeficacia contribuye a un mejor manejo del estrés y al desempeño clínico^(20)^, dichas prácticas pueden favorecer el equilibrio emocional, aunque se necesitan nuevos estudios para confirmar su efectividad. El apoyo social se destacó como un factor protector, especialmente cuando está mediado por la participación activa de los educadores^(23,24)^. Se señalaron estrategias colectivas, como grupos de discusión, *debriefings* y espacios institucionales de diálogo, como medidas deseables para compartir experiencias, fortalecer los vínculos y reducir el estrés.

El manejo del estrés comienza con la preparación de los estudiantes para la práctica clínica, mediante medidas que reduzcan la ansiedad y desarrollen habilidades. Los cursos que aborden las causas y los efectos del estrés y fortalezcan la autoconfianza, la autoeficacia y la resiliencia son fundamentales para enfrentar los desafíos clínicos^(20,25)^. En este sentido, los resultados refuerzan la necesidad de iniciativas estructuradas para la preparación clínica, el manejo del estrés y la promoción del bienestar, incluyendo programas educativos, tutorías, simulaciones realistas y actividades de regulación emocional^(15,26,27,28,29)^. Los programas basados en la atención plena (*mindfulness*) y las redes de apoyo social también demuestran beneficios relevantes^(30)^. En el ámbito organizacional, los esfuerzos conjuntos entre instituciones educativas, servicios de salud, educadores y preceptores son fundamentales para crear entornos de práctica acogedores y comprometidos con la formación en enfermería, reduciendo los factores estresantes y favoreciendo el aprendizaje^(8)^. El uso de sesiones de reflexión al final de las actividades prácticas se destaca como una estrategia relevante para la evaluación, el feedback y el perfeccionamiento del proceso formativo.

En conjunto, los hallazgos de este estudio indican que el estrés es experimentado de manera individual por los estudiantes, pero es generado, intensificado o atenuado por factores colectivos, relacionales e institucionales, configurándose como un fenómeno coproducido dentro del contexto formativo.

El estrés inicial no surge únicamente “desde dentro” del estudiante, sino que se construye a partir de condiciones concretas de la práctica clínica, como el inicio abrupto de las actividades, la carga de tareas y la forma en que se organiza la pasantía, articuladas con las relaciones interpersonales establecidas en los escenarios de aprendizaje, especialmente con la actuación del educador y los significados previamente atribuidos a la práctica a través de los relatos de estudiantes veteranos.

Además, la dimensión colectiva de la formación influye en la forma en que se percibe y se maneja el estrés, teniendo en cuenta la presencia o ausencia de espacios institucionales de escucha, el apoyo entre pares y las posibilidades de compartir experiencias. Esta comprensión amplía la interpretación del estrés en la práctica clínica como un proceso dinámico, influenciado por las condiciones formativas, y contribuye a reflexionar sobre cómo los diferentes arreglos pedagógicos e institucionales pueden repercutir en la adaptación de los estudiantes a lo largo de la formación en enfermería.

## CONCLUSIÓN

Este estudio permitió comprender que los estudiantes de enfermería viven la práctica clínica como un momento formativo marcado por la inseguridad, la incomodidad y el estrés, especialmente al inicio de las actividades, cuando aún no están tan familiarizados con el entorno, las exigencias de la atención y las relaciones que se establecen en los entornos de práctica. Al explorar las experiencias y percepciones de los estudiantes, los resultados evidencian que este estrés no se manifiesta de manera homogénea, sino que está determinado por las condiciones pedagógicas, relacionales e institucionales en las que se desarrolla la práctica clínica.

La identificación de estrategias individuales y colectivas de enfrentamiento amplía la comprensión sobre cómo los estudiantes lidian con los desafíos de la práctica clínica y pone de manifiesto que el manejo del estrés puede favorecer procesos de adaptación más seguros a lo largo de la formación. En este sentido, el estudio contribuye al demostrar que el estrés percibido no constituye un obstáculo fijo o inevitable, sino una experiencia que puede reconocerse y gestionarse mediante acciones educativas, apoyo social y una organización institucional más sensible a las necesidades de los estudiantes.

Al integrar estas dimensiones, los hallazgos ofrecen fundamentos para mejorar las prácticas pedagógicas e institucionales que enriquezcan la experiencia clínica, favorezcan la permanencia y el desarrollo de los estudiantes y respalden su trayectoria formativa a lo largo de las diferentes etapas de la formación en enfermería. Además, los hallazgos pueden orientar investigaciones futuras, especialmente estudios longitudinales que evalúen el impacto de las intervenciones educativas sobre el estrés a lo largo de la formación en enfermería.

## Data Availability

Todo el conjunto de datos que respalda los resultados de este estudio se publicó en el propio artículo.
